# Synthetic Lethal Drug Combinations Targeting Proteasome and Histone Deacetylase Inhibitors in TP53-Mutated Cancers

**Published:** 2020

**Authors:** Shaoli Das, Xiang Deng, Kevin Camphausen, Uma Shankavaram

**Affiliations:** Radiation Oncology Branch, National Cancer Institute, National Institutes of Health, Bethesda, Maryland, USA

**Keywords:** Synthetic lethality, In-silico analysis, Tumor genome atlas, Drug sensitivity, TP53-mutation, Proteasome, Histone deacetylase

## Abstract

**Background:**

We have recently published SL-BioDP, a web resource for querying, exploration and visualization of potential synthetic lethal targets and possible synergistic drug combinations for 18 cancer types.

**Methods:**

From our predictive synthetic lethality model used in SL-BioDP, we inferred TP53 mutation lead to potential synergistic drug combination of Bortezomib and Vorinostat. Here we show, how to extrapolate the drug combination results by combining drug screening data from cancer cell lines and showed the potential synergy of the drug targets, proteasome, and histone deacetylase (HDAC) pathways respectively, for patient survival advantage.

**Results:**

We found that TP53 mutation is potentially synthetic lethal with multiple genes from the proteasome and HDAC pathways exclusively in many cancer types. Also, HDAC and proteasomes were found to have potential synthetic lethal relationship. Using drug screening data in cancer cell line, the sensitivity of the HDAC inhibitor drug Vorinostat was found to be increased in TP53 mutated cells where the proteasome pathway was downregulated.

**Conclusions:**

Our in-silico pharmacogenomic study indicates that the potential synergistic drug combination of proteasome and HDAC inhibitors may be considered as potential treatment for TP53-mutant cancers.

## Introduction

Tumors harboring mutations in certain oncogenes are often dependent on activation of certain pathways which becomes essential for the survival of the cancer cells. This condition is formally known as synthetic lethality, a state when simultaneous loss of two genes is lethal to a cancer cell, while the loss of the individual genes is not. These alternative genes or pathways are of potential interest as drug targets, which makes it possible to uniquely target tumor cells harboring oncogenic mutations [1]. Also, targeting the interaction partner of an undruggable cancer gene provides an alternative way for treating cancer patients with mutation in that gene. Using the multi-omic tumor data from The Cancer Genome Atlas (TCGA) [2], we recently developed an algorithm for screening potential synthetic lethal partners, based on mutations in cancer genes [3]. We extended our analysis for searching SL interactors for frequently mutated cancer genes in 18 tumor types and developed a comprehensive web resource SL-BioDP to enable query and visualization of alternative drug targets for treating cancers harboring mutations in certain genes and assess the clinical relevance [4]. We also used our predicted SL interactions to infer potential synergistic drug combinations that would target both SL partners for effective killing of cancer cells. Examples of such drug combinations that has proven to be effective clinically include the combination of BRAF inhibitor dabrafenib and MEK inhibitor trametinib, which has been approved for treatment of melanoma[5].

In this note, we explore the synergistic drug combinations of the proteasome inhibitor drug Bortezomib and the histone deacetylase (HDAC) inhibitor drug Vorinostat in context of TP53 mutation in cancers. Previously, synergistic combination of Bortezomib and Vorinostat has been tested for treating mantle cell lymphomas [6]. On the other hand, previous literatures have linked mutation in the tumor suppressor gene TP53 with activation of proteasome subunits for adaptation to proteotoxic stress and evasion of apoptosis in cancer cells [7], leading to the idea that targeting proteasome pathway may be a potential treatment strategy for tumors harboring p-53 mutation. Using our SL prediction algorithm combined with cancer patient survival analysis from TCGA clinical data and in-silico drug sensitivity analysis from cancer cell lines, we show results supporting the idea that the combination of the drugs Bortezomib and Vorinostat might be beneficial for treating TP53-mutated cancers.

## Materials and Methods

Potential synthetic lethal candidates for TP53 mutation in cancers were selected from the SL-BioDP database with the following criteria: predicted SL score greater than 0.7 and overall survival p-value <0.05 (TP53 mutated, SL gene down vs up) [4]. To assess the clinical outcome of under-expression vs. over-expression of the predicted SL gene in cases with mutation in the primary gene (here TP53), Kaplan Meier survival analysis was performed on overall free survival in TCGA clinical data.

To assess the potential sensitivity of a drug, depending on mutation in TP53, a p-value is calculated using t-test on the LNIC50 values between TP53 mutated vs non-mutated cancer cells from the Genomics of Drug Sensitivity in Cancer (GDSC) project data [8]. Single sample enrichment scores for proteasome or HDAC pathways across 165 cancer cell lines from GDSC was calculated using ssGSEA analysis from R package GSVA [9]. RMA normalized microarray gene expression data from GDSC was used for ssGSEA analysis. To assess the alteration of drug sensitivity depending on low enrichment of a pathway, the cell lines are stratified into two sets based on the ssGSEA scores of the pathway. Cell lines with pathway ssGSEA z-score < −2 were considered to have low enrichment of that pathway. The difference between drug IC50 in cell lines with low ssGSEA scores (z-score < −2) with the rest of cell lines was calculated using a one-sided t-test with an alternative hypothesis equal to greater.

## Results

**Figure 1:**
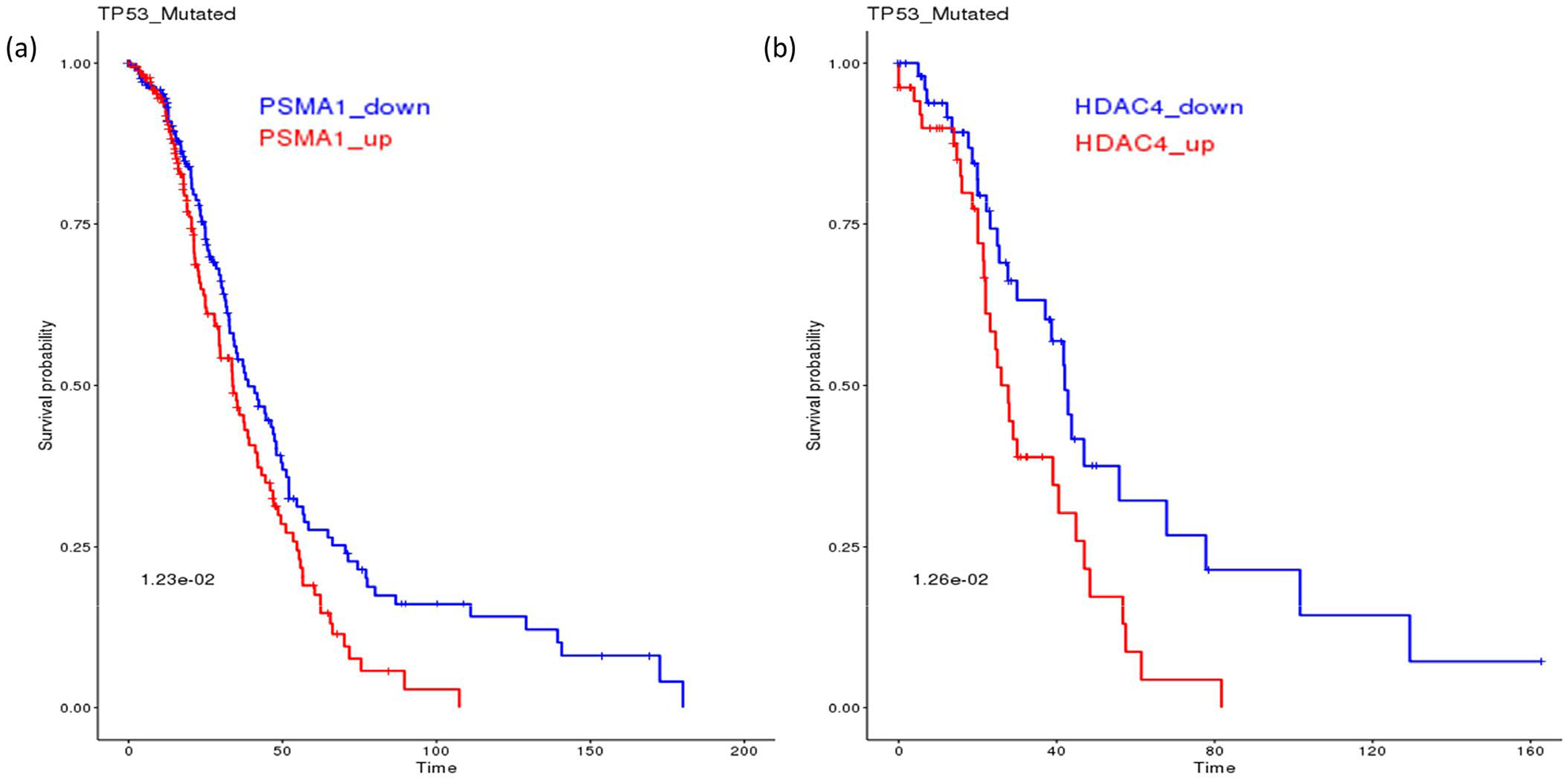
The effect of TP53 mutation on SL genes from proteasome and HDAC pathways. (a) In TP53 mutated head and neck cancer patients from TCGA, downregulation of the proteasome gene PSMA1 is associated with better survival. (b) In TP53 mutated lung adenocarcinoma patients from TCGA, downregulation of HDAC4 is associated with better survival.

Searching for potential synthetic lethal partners for TP53 mutation from the SL-BioDP identified multiple genes from the proteasome pathways as candidate SLs, e.g. PSMA6 and PSMC6 in the breast cancer (BRCA), PSMD3, PSMC4 in bladder cancer (BLCA), PSMA3 in cervical cancer (CESC), PSMG1 in liver cancer (LIHC), PSMD10 in lung adenocarcinoma (LUAD), PSMB1 in ovarian cancer (OV), PSMB4 in pancreatic cancer (PAAD), PSMD7 in kidney renal cancer (KIRC), PSMD1 and PSMC3 in melanoma (SKCM), PSMC1, PSMA5 and PSMA1 in head and neck cancer (HNSC). On the other hand, multiple genes from the HDAC pathways were also shortlisted as potential SL partners for TP53 mutation, e.g. HDAC8 in BRCA, HDAC5, HDAC6 and HDAC7 in BLCA, HDAC2 and HDAC6 in lower grade glioma (LGG), HDAC4 in LUAD. Figure 1 a and b shows examples of potential clinical benefit of downregulation of PSMA1 in TP53 mutated HNSC patients and HDAC4 in TP53 mutated LUAD patients, using Kaplan-Meier analysis on TCGA clinical data.

**Figure 2:**
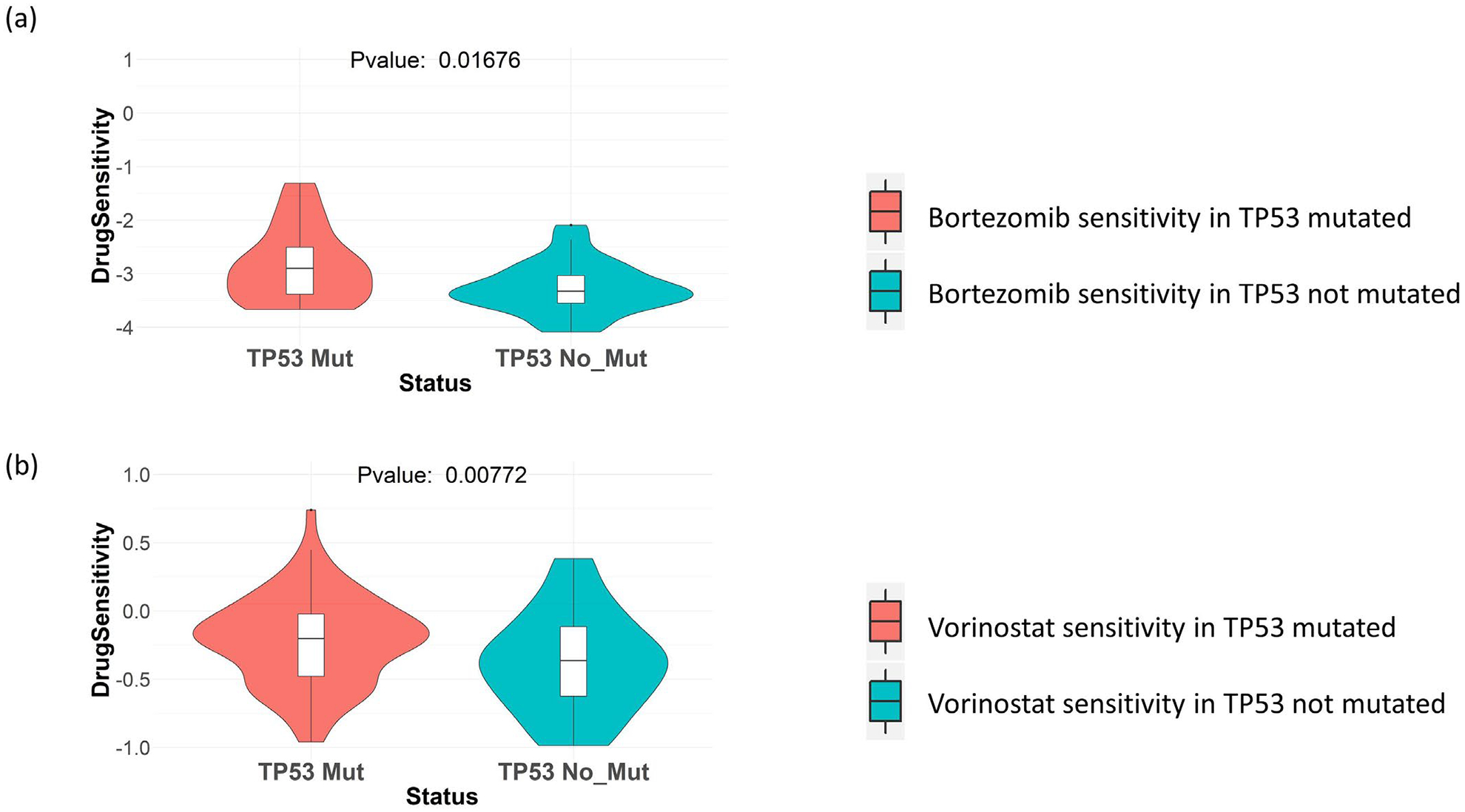
Drug sensitivity of proteasome and HDAC inhibitors in GDSC cell lines. TP53 mutation in cancer cell lines from the primary sites breast, ovary, kidney, prostate and salivary gland, is shown to be associated with increased sensitivity to **(a)** proteasome inhibitor drug Bortezomib. **(b)** HDAC inhibitor drug Vorinostat.

Next, we checked the effect of TP53 mutation on the sensitivity towards drugs targeting proteasome pathway and HDAC pathway. From drug screening data in cancer cell lines collected from GDSC, we saw that TP53 mutated cancer cell lines (breast, ovary, kidney, prostate and salivary gland cancer cell lines) show better sensitivity to the proteasome inhibitor drug Bortezomib, as well as HDAC inhibitor drug Vorinostat (Figures 2a and 2b). These results indicate that both proteasome inhibitors and HDAC inhibitors can be possible SL-based drugs for treating TP53 mutated tumors.

**Figure 3:**
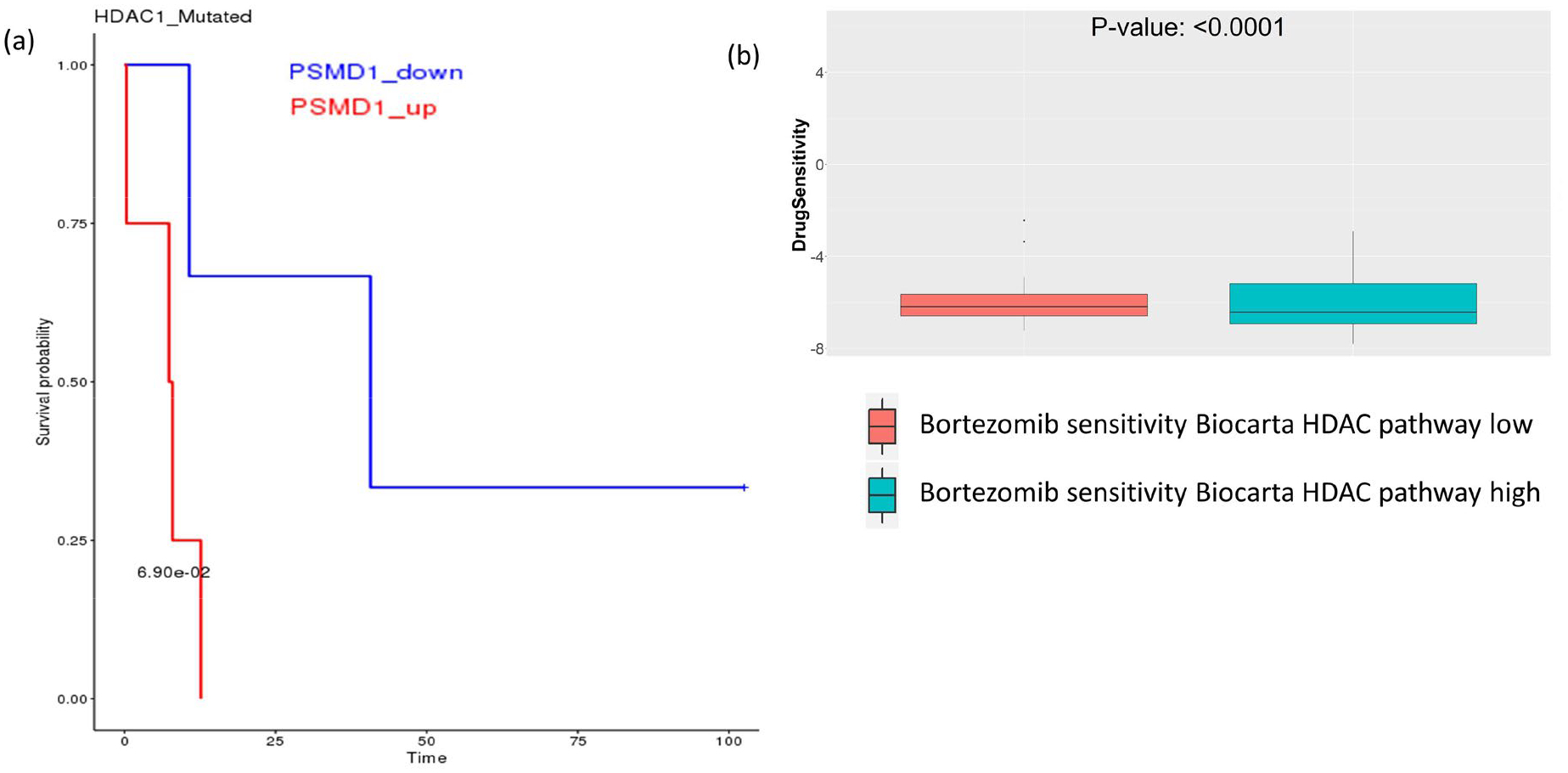
Survival analysis and drug sensitivity showing the effect of downregulation of proteasome and HDAC pathway genes. **(a)** In HDAC mutated breast cancer patients from TCGA, downregulation of the proteasome family gene PSMD1 is associated with better survival. **(b)** In GDSC drug screening data, proteasome inhibitor drug Bortezomib is associated with better sensitivity in cell lines having low enrichment of HDAC pathway.

On the other hand, we tried to look at the possible SL relationship between HDACs and proteasomes. From SL-BioDP, we found HDAC1 has a potential SL relationship with some members of the proteasome family, e.g. PSMA2, PSMD1 and PSMF1 (Figure 3a shows survival benefit of HDAC1 mutated BRCA patients with downregulation of PSMD1). Checking the drug sensitivity data, we found that enrichment of the HDAC pathway has significant effect on the sensitivity to proteasome inhibitor Bortezomib (Figure 3b). This indicates towards possible synergistic combinations of the proteasome inhibitors and HDAC inhibitors.

**Figure 4:**
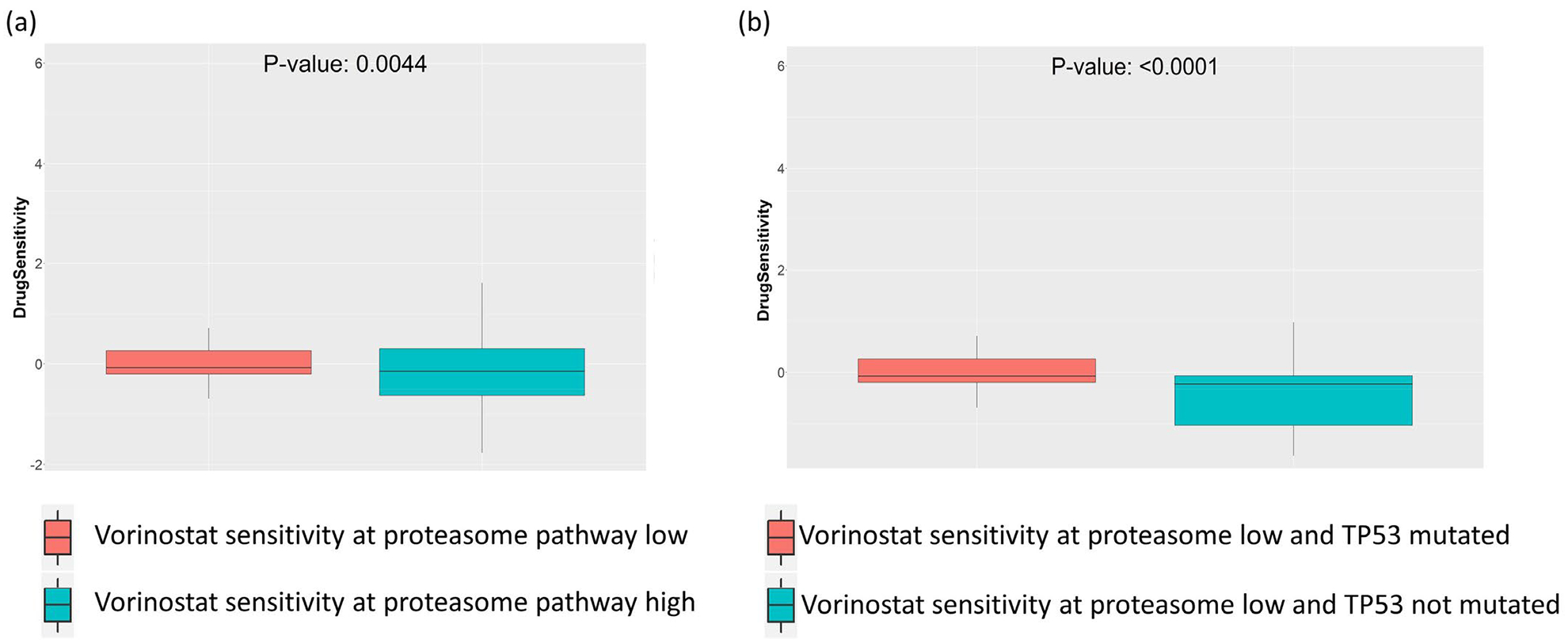
Synthetic lethal drug sensitivity of proteasome or HDAC pathway inhibitors in GDSC cell lines with TP53 mutation. **(a)** HDAC inhibitor drug Vorinostat is associated with better sensitivity in cell lines having low enrichment of proteasome pathway. **(b)**, HDAC inhibitor drug Vorinostat is associated with better sensitivity in cell lines with low enrichment of proteasome pathway.

Next, we tried to see what the effects of altered enrichment of the proteasome pathway on the HDAC inhibitor Vorinostat would be when TP53 is mutated. For this analysis, we selected the TP53 mutated cancer cell lines from GDSC drug screening data, and then performed a t-test on this set of cell lines stratified by the enrichment of the proteasome pathway. We observed that in TP53 mutated cancer cell lines, low enrichment of the proteasome pathway was associated with higher sensitivity to Vorinostat (Figure 4a). Conversely, we checked the effect of TP53 mutation on the sensitivity to Vorinostat, in cell lines where proteasome pathway is downregulated. Here we selected the cell lines where enrichment of proteasome pathway was low (enrichment score < −2), and then performed t-test on Vorinostat sensitivity in cell lines stratified by TP53 mutation. We again saw increased sensitivity to Vorinostat in TP53 mutated cell lines where proteasome pathway was less enriched (Figure 4b).

## Discussion

Harnessing synthetic lethal relationship between cancer genes and pathways holds huge promise for discovering new treatment strategies involving single or multi-drug combinations for cancer patients with certain oncogenic mutations. Our published web-tool SL-BioDP has been developed to facilitate such discoveries based on integrating tumor genomic, clinical and drug data in a single platform using a computational algorithm that enables prioritizing candidate drugs for treating tumors based on the mutation profile. Here we discuss a potential treatment option for tumors harboring mutation in TP53 gene.

Using predictions from SL-BioDP, we saw that mutation in the tumor suppressor gene TP53 makes it synthetic lethal to the genes from proteasome pathways as well as HDACs. Using drug screening data from cancer cell lines, we observed that TP53 mutation is associated with increased sensitivity to both proteasome inhibitor drug Bortezomib and HDAC inhibitor drug Vorinostat. HDAC and proteasome pathways also showed potential synthetic lethal relationship and low enrichment of HDAC pathway were seen to be associated with greater sensitivity to proteasome inhibitor Bortezomib. Moreover, in our in-silico analysis on TP53 mutated cancer cell lines, low enrichment of proteasome pathway was seen to increase the sensitivity to Vorinostat treatment. Conversely, in cell lines with low enrichment of proteasome pathway, TP53 mutation was associated with greater sensitivity to Vorinostat compared to TP53 wild-type cells. Taken together, our in-silico analysis indicates towards possible synergistic combination of proteasome and HDAC inhibitors as a treatment option for tumors harboring TP53 mutation.

The results from our in-silico analysis on the potential usefulness of proteasome or HDAC inhibitors in TP53-mutated tumors, as well as the combination of proteasome and HDAC inhibitors is supported by the previous literature. TP53 mutation in tumors is associated with activation of the proteasome pathways, that help tumors to evade apoptosis by adapting to proteotoxic stress[7]. Conversely, HDAC inhibitors have been shown to exert cytotoxicity preferentially in TP53-mutant cancer cell lines [10,11], though this observation is controversial as there have been contradicting reports suggesting different HDAC inhibitors may have different effects on TP53 mutant or wild-type cells [12]. Nonetheless, from multiple publications there is an indication towards the effectiveness of proteasome inhibition or HDAC inhibition in TP53-mutated tumors [10,11,13]. On the other hand, the combination of proteasome and HDAC inhibitors has been shown to interact strongly to promote apoptosis in cancer cell lines [14–16], and this combination therapy has been tested in clinical trials for mantle cell lymphomas [6]. Our observation from the in-silico study that this combination might work preferentially in TP53-mutated tumors is also supported by a recent literature that shows treatment with proteasome inhibitors induced cell death in gynecologic cancer cell lines harboring gain-of-function TP53 mutation, and addition of HDAC inhibitors further enhanced this effect [17]. However, these are preliminary observations from cancer cell line data, and the true effectivity of this combination treatment for TP53-mutant cancers need further in-depth experimentation before they can be considered for clinical trial.

## Conclusion

Our in-silico analysis indicates that the combination of proteasome inhibitor drug Bortezomib and HDAC inhibitor drug Vorinostat can be potentially beneficial for TP53-mutated tumors.
